# Initial Progress Toward Development of a Voice-Based Computer-Delivered Motivational Intervention for Heavy Drinking College Students: An Experimental Study

**DOI:** 10.2196/mental.7571

**Published:** 2017-06-28

**Authors:** Christopher W Kahler, William J Lechner, James MacGlashan, Tyler B Wray, Michael L Littman

**Affiliations:** ^1^ Center for Alcohol and Addiction Studies Department of Behavioral and Social Sciences Brown University School of Public Health Providence, RI United States; ^2^ Brown University Computer Science Department Providence, RI United States

**Keywords:** Computer-delivered intervention, voice-based systems, alcohol intervention, heavy drinking

## Abstract

**Background:**

Computer-delivered interventions have been shown to be effective in reducing alcohol consumption in heavy drinking college students. However, these computer-delivered interventions rely on mouse, keyboard, or touchscreen responses for interactions between the users and the computer-delivered intervention. The principles of motivational interviewing suggest that in-person interventions may be effective, in part, because they encourage individuals to think through and speak aloud their motivations for changing a health behavior, which current computer-delivered interventions do not allow.

**Objective:**

The objective of this study was to take the initial steps toward development of a voice-based computer-delivered intervention that can ask open-ended questions and respond appropriately to users’ verbal responses, more closely mirroring a human-delivered motivational intervention.

**Methods:**

We developed (1) a voice-based computer-delivered intervention that was run by a human controller and that allowed participants to speak their responses to scripted prompts delivered by speech generation software and (2) a text-based computer-delivered intervention that relied on the mouse, keyboard, and computer screen for all interactions. We randomized 60 heavy drinking college students to interact with the voice-based computer-delivered intervention and 30 to interact with the text-based computer-delivered intervention and compared their ratings of the systems as well as their motivation to change drinking and their drinking behavior at 1-month follow-up.

**Results:**

Participants reported that the voice-based computer-delivered intervention engaged positively with them in the session and delivered content in a manner consistent with motivational interviewing principles. At 1-month follow-up, participants in the voice-based computer-delivered intervention condition reported significant decreases in quantity, frequency, and problems associated with drinking, and increased perceived importance of changing drinking behaviors. In comparison to the text-based computer-delivered intervention condition, those assigned to voice-based computer-delivered intervention reported significantly fewer alcohol-related problems at the 1-month follow-up (incident rate ratio 0.60, 95% CI 0.44-0.83, *P*=.002). The conditions did not differ significantly on perceived importance of changing drinking or on measures of drinking quantity and frequency of heavy drinking.

**Conclusions:**

Results indicate that it is feasible to construct a series of open-ended questions and a bank of responses and follow-up prompts that can be used in a future fully automated voice-based computer-delivered intervention that may mirror more closely human-delivered motivational interventions to reduce drinking. Such efforts will require using advanced speech recognition capabilities and machine-learning approaches to train a program to mirror the decisions made by human controllers in the voice-based computer-delivered intervention used in this study. In addition, future studies should examine enhancements that can increase the perceived warmth and empathy of voice-based computer-delivered intervention, possibly through greater personalization, improvements in the speech generation software, and embodying the computer-delivered intervention in a physical form.

## Introduction

In the United States, heavy drinking among college students is a major public health concern that results in negative consequences for both drinking and nondrinking students [1]. The well-developed literature shows that brief, single-session interventions can reduce a variety of problematic drinking outcomes in college students [2-6]. Among the most well studied of these interventions are those based on principles of motivational interviewing (MI) [7], and interventions utilizing MI principles appear to have the largest effects on drinking outcomes [6]. More recently, some components of MI-based brief interventions have been adapted for delivery by computer. Evidence suggests that students receiving computer-delivered interventions reduce problematic drinking [3], which can reduce costs and improve dissemination compared to more traditional face-to-face interventions. However, meta-analyses exploring the effects of computer-delivered and face-to-face interventions across studies show that face-to-face interventions may produce longer-lasting effects than computer-delivered interventions [8,9], and that face-to-face interventions may outperform computer-delivered interventions in their impact on drinking quantity, peak blood alcohol content, and alcohol-related problems [10-16]. Together, these studies suggest that computer-delivered interventions may be useful tools for helping college students build motivation to change their drinking, but that they fall short of face-to-face interventions in some important ways.

A central tenet of MI, supported by research, is that the elicitation of “change talk” (ie, verbal behavior that is supportive of behavior change) is a key active ingredient of the intervention that predicts later changes in behavior [17-20]. This change talk is elicited in face-to-face MI interventions through open-ended questions and reflective listening techniques (including simple reflections, paraphrased reflections, double-sided reflections, and summarizations) that allow clients to hear their own change talk. MI process research shows that clients are, in fact, significantly more likely to engage in change talk directly following simple reflections, complex reflections, and open questions posed by the interventionist [21]. Although existing computer-delivered interventions can elicit information from users and provide personalized feedback, their capacity to utilize complex reflections and open-ended questions effectively may be limited. Furthermore, existing computer-delivered interventions rely on a personal computer (PC) keyboard, mouse, or touchscreen to capture participant’s responses that lack the capacity to allow users to speak aloud and to hear their own change talk, which may be an important factor in the success of MI interventions. These limitations in existing computer-delivered interventions could be mitigated by allowing voice-based interaction between the human user and the computer-delivered intervention. In a voice-based system, users could respond to open questions about their behaviors and attitudes with natural language, and the computer-delivered intervention could use reflective listening techniques to encourage deeper reflection and highlight discrepancies between current behavior and desired goals.

There are numerous challenges to developing a voice-based computer-delivered intervention that mirrors the processes occurring in human-delivered MI more closely than existing computer-delivered interventions. Although it is relatively straightforward to program open-ended prompts for a computer to deliver using speech software and although natural language recognition programs are becoming increasingly sophisticated [22-24], understanding the meaning of the users’ speech in response to open questions is a far greater challenge [25,26]. Making a conversation with a computer-delivered intervention feel natural and empathic requires substantial development efforts. Nonetheless, the questions and prompts used in MI follow some prototypical forms, and users’ responses to specific questions are likely to fall within a relatively limited and definable set of topics [27]; the limited universe of potential questions and content of responses could make feasible the development of a voice-based computer-delivered intervention that responds appropriately to users [28].

The purpose of this project was to take initial steps toward development of a voice-based computer-delivered intervention by creating a system of questions and responses that would mirror the content and style of a brief MI. For this initial development, we chose to create a “Wizard of Oz” computerized system where participants would speak directly to a computer screen and a human controller would select appropriate responses and follow-up questions from an onscreen menu, which would then be “spoken” by the computer using voice-generation software. Thus, our software was responsible for answer generation and speech synthesis, and a human operator handled the problem of speech understanding and dialog flow. Because automating these features will require significant engineering work, we focused on the proof of concept as demonstrated by this mixed human/computer approach. The system was designed to ask open-ended questions, encourage deeper reflection of motivations, and provide MI-consistent responses such as paraphrased reflections, double-sided reflections, affirmations, and summary statements.

We tested the feasibility and acceptability of our human-controlled version of a voice-based computer-delivered intervention with a sample of heavy drinking college students. We examined (1) participants’ ratings of how well the voice-based computer-delivered intervention attained key goals of MI, such as understanding the participant, being nonjudgmental, and being empathic and engaging; (2) whether participants were willing to set a goal to change drinking during the interaction; and (3) whether participants accepted a printed sheet on tips for reducing drinking at the end of the session. We also conducted a follow-up assessment with participants 1 month after the initial interaction with the voice-based computer-delivered intervention in order to test our primary hypotheses that participants receiving the voice-based computer-delivered intervention would report a significant increase in perceived importance of changing their drinking and report significant reductions in drinking and alcohol-related problems, consistent with the literature on computer-delivered interventions in college student populations. In order to gauge in a preliminary manner how the voice-based computer-delivered intervention might differ in its effect from traditional text-based computer-delivered intervention, we randomized one-third of participants to a text-based computer-delivered intervention, which matched the voice-based computer-delivered intervention in content, but relied on mouse and keyboard entries of participant responses and provided only text-based responses from the computer. We compared the voice-based computer-delivered intervention to the text-based computer-delivered intervention on acceptability measures. We also examined the drinking outcomes of participants assigned to the voice-based computer-delivered intervention versus the text-based computer-delivered intervention at 1-month follow-up. Given the literature cited previously regarding the importance of change talk and our supposition that a voice-based computer-delivered intervention may increase processing of change talk through verbalization, we hypothesized that the voice-based computer-delivered intervention, compared to the text-based computer-delivered intervention, would result in greater increases in perceived importance of changing drinking and greater reductions in drinking behavior and related problems. These secondary hypotheses were considered preliminary because the study was not fully powered to assess differences between conditions over time, and our emphasis was on the overall direction of effects across measures.

## Methods

### Participants

Participants were recruited from local colleges and universities using flyers and Web-based advertisements. Eligible participants were enrolled in undergraduate or graduate programs in the Northeastern United States, were 18 years of age or older, and endorsed at least one episode of heavy drinking (≥5 drinks in a single sitting for men, ≥4 drinks for women) in the past 30 days.

### Power and Sample Size Determination

Sample size was determined by taking the following considerations into account. First, we wanted ample power to detect—within the voice-based computer-delivered intervention condition—significant changes in importance of changing drinking and drinking-related outcomes, our primary hypotheses. An initial sample size of 60, assuming an 85% follow-up rate, provided power of .94 to detect a medium effect size of *d*=0.50 and power of .80 to detect a somewhat smaller effect size of *d*=0.40; this power was determined to be adequate for the primary hypotheses. We also wanted to acquire a large enough body of verbal participant responses and human-controller response selections to facilitate future machine-learning approaches to approximate the decisions that human controllers made (results not described here). Results of machine-learning approaches could be used as the initial seeds for developing a fully automated voice-based computer-delivered intervention, and we decided that having 60 completed sessions should provide a minimal level of data to initiate that work. Given resource limitations, we were not able to randomize 60 participants to the text-based computer-delivered intervention using a 1:1 allotment. Therefore, we used a 2:1 randomization scheme, with 60 participants randomized to the voice-based computer-delivered intervention and 30 to the text-based computer-delivered intervention. Those sample sizes provided only power of .60 for an effect size of *d*=0.50 for between-groups differences; power was .80 to detect an effect size of *d*=0.63.

### Procedure

All procedures were approved by the Brown University Institutional Review Board. Following eligibility assessment by telephone, those who appeared eligible were invited for a baseline session, which occurred in the laboratory. At the baseline interview, participants first completed written informed consent followed by measures of demographics, alcohol use over the past 30 days, alcohol-related problems, and importance of changing drinking. Breath alcohol concentration was measured at baseline; those with values greater than zero were asked to reschedule. After baseline assessments were completed, participants were randomized in a 2:1 ratio using the urn procedure [29,30]—to ensure equal balancing on gender and number of heavy drinking days—to one of two experimental interventions: (1) a human-controlled voice-based computer-delivered intervention with computer-generated voice communication or (2) a computer-based text-and-click entry interface comparison condition.

**Figure 1:**
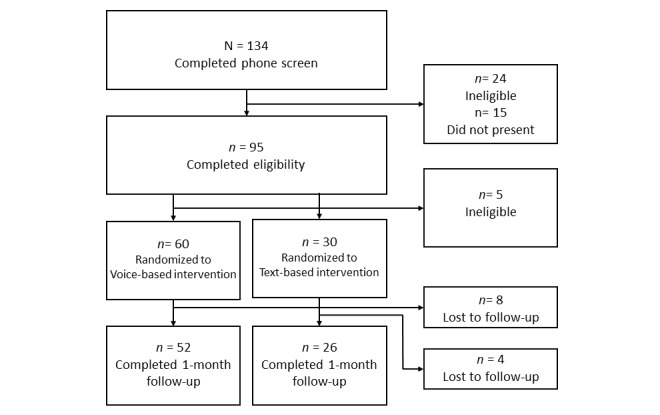
Participant flow.

Thirty days after the baseline session, email links were sent with instructions to complete follow-up surveys. (See Figure 1 for participant flow through follow-up.) Participants were paid US $20 for completing the baseline appointment and US $30 for completing the follow-up assessment.

### Measures

Alcohol use over the past 30 days was assessed using an online timeline follow-back measure [31], which assessed the number of standard drinks (12 oz beer, 5 oz wine, 1-1.5 oz liquor as a “shot” or in a mixed drink) participants consumed each day over the past 30 days and the approximate number of hours over which these drinks were consumed. Alcohol-related problems were assessed using the Brief Young Adult Alcohol Consequences Questionnaire (BYAACQ) [32]. Dichotomous items (yes/no) were summed for a total number of alcohol-related consequences experienced in the past month. The BYAACQ has been demonstrated to be sensitive to changes in alcohol use over time [33] and has shown high internal consistency in research with college students (alpha=.89 [32]). The importance of changing drinking was assessed with a single item (“How important is it to change your drinking?”), which was rated on a 0=“not at all important” to 10=“extremely important” scale; this measure has been used previously in college-aged samples [34] and has shown to be predictive of change in alcohol use behaviors in prospective analyses [34]. Each of these measures was also collected online at follow-up, 30 days after the baseline and brief intervention.

To assess the extent to which the system approximated MI counseling characteristics, we administered two brief surveys, specifically designed for this project, to all participants that contained (1) five items (7-point Likert scale: 1=“not at all” to 7=“very”) reflecting general therapist traits (eg, warmth, understanding) as well as (2) eight items (4-point Likert scale: 1=“strongly disagree” to 4=“strongly agree”) reflecting MI strategies (eg, helped me to talk about my ideas for change). Both the five-item scale assessing general therapist traits and the eight-item scale assessing MI strategies demonstrated good internal consistency (Cronbach alpha=.89 and .83, respectively).

Other relevant measures were assessed from within the intervention as participants completed it, including whether participants (1) set a goal for reducing their drinking and/or (2) agreed to receive further information on changing their drinking.

### Brief Motivational Intervention Computerized Delivery Systems

The computer-delivered interventions contained several common facets. They both assessed users’ levels of drinking and provided feedback in the form of peer-based norms. The computer-delivered interventions assessed positive and negative consequences of drinking, and used 0 to 10 rulers to assess participants’ perceived importance of and confidence in changing drinking behavior, followed by assessment of reasons for those ratings. Finally, if participants endorsed willingness, the computer-delivered interventions assisted users in setting a goal for change. Additional information (a pamphlet) on reducing drinking was also offered to users at the end of the session.

Participants assigned to the text-based computer-delivered intervention completed the session with no observer, interacting with the system by entering their responses using a keyboard and mouse. For example, the system presented an onscreen question asking what the user liked about drinking, and the participant responded by viewing a list of possible options and checking the corresponding boxes that applied to their experience. The system then reflected the positive and negative consequences endorsed by the participant in text presented on the computer screen.

Participants assigned to the voice-based computer-delivered intervention completed the intervention by speaking to the system. Their verbal responses were captured by a microphone in the interview room and were monitored by a research assistant outside of the room, who could also see the participant through a one-way mirror. The research assistant listened to the questions that the system asked and based on a participant’s responses selected appropriate paraphrases of content or prompts to the participant for further information from a pre-established list of possible responses. For example, the system verbally asked what the user liked about drinking and, as the user responded verbally, the human controller checked off responses such as drinking “helps you have fun” or that drinking “tastes good.” The positive and negative consequences of drinking were then verbally reflected to the participant via computerized voice, with the phrases strung together to create a double-sided reflection: “On the one hand you like that drinking..., but on the other hand, you do not like that...” The voice-based computer-delivered intervention also allowed custom user responses to be entered and allowed the human controller to have the system inject common follow-up questions and comments, such as “Can you repeat that?” and “What else?” The voice used to speak the computer responses was selected from the standard speech-to-text voice options available on Mac OS X.

### Statistical Analysis

We first examined participants’ ratings of the characteristics of the voice-based computer-delivered intervention to determine how well the system met the objective of reflecting positive therapist traits (eg, how supportive was the system) and MI-based therapy traits (eg, how well did the system help you talk about your own reasons for change). We compared these ratings to those given to the text-based computer-delivered intervention using *t* tests. We also examined participants’ willingness to set a goal related to reducing alcohol consumption and to take additional information on how to limit alcohol use at the end of the session; we used chi-square tests to compare the proportion of participants setting goals and accepting information in the voice-based computer-delivered intervention and text-based computer-delivered intervention conditions. We next examined follow-up data, starting with an examination of how those completing follow-ups differed from those not completing follow-ups. We then conducted paired *t* tests to test the hypothesis that participants receiving voice-based computer-delivered intervention would show significant increases from baseline to the 1-month follow-up in perceived importance of changing drinking and confidence in their ability to change drinking and would show significant decreases in drinking and alcohol-related problems. To test our secondary hypotheses regarding differences between the voice-based and text-based computer-delivered interventions, we conducted linear regressions to test the effects of experimental condition on self-rated importance of changing drinking and confidence in ability to change drinking, as well as number of drinks consumed per week at the 1-month follow-up. Both number of heavy drinking days and number of alcohol-related problems (BYAACQ) represented count data and therefore were analyzed with a negative binomial distribution and logit link function. For each regression model, the experimental condition was dummy-coded as the primary independent variable with text-based computer-delivered intervention as the reference group; gender and the respective baseline assessment of the dependent variable were entered as covariates.

## Results

### Preliminary Analyses

Demographic characteristics of the 90 participants in the study are shown in Table 1, broken down by experimental condition.

### System Traits

General therapist traits were rated at the midpoint between “not at all” and “very” for the voice-based computer-delivered intervention group, and participants agreed that the voice-based computer-delivered intervention system was consistent with MI counseling style (mean 3.0). Within the voice-based computer-delivered intervention condition, 61% (37/60) of participants were willing to set a goal to reduce their drinking, and 60% (36/60) accepted additional information on reducing their drinking at the conclusion of the session. As shown in Table 2, there were no significant differences in ratings of therapist and MI traits between the voice-based computer-delivered intervention and text-based computer-delivered intervention conditions. Similarly, chi-square analyses revealed no significant difference between conditions on willingness to set a goal (voice-based intervention: 36/59, 61%; text-based intervention: 18/30, 60%) or take additional information (voice-based intervention: 36/60, 60%; text-based intervention: 14/29, 48%), respectively, at the end of the session.

**Table 1:** Demographics for the full sample and intervention.

Variable		Total (N=90)	Computer-delivered intervention	
			Voice (n=60)	Text (n=30)
Age (years), mean (SD)		21.6 (2.8)	21.7 (2.3)	21.47 (3.5)
**Gender, n (%)**				
	Female	51 (57)	32 (53)	19 (63)
	Male	38 (42)	27 (45)	11 (37)
	Other	1 (1)	1 (2)	0 (0)
**Race, n (%)**				
	Asian	12 (13)	8 (13)	4 (13)
	Black	13 (14)	12 (20)	1 (3)
	Biracial	1 (1)	1 (2)	0 (0)
	Multiracial	5 (6)	3 (5)	2 (7)
	Other race	5 (6)	3 (5)	2 (7)
	Pacific Islander	1 (1)	0 (0)	1 (3)
	White	53 (59)	33 (55)	20 (67)
Years of education, mean (SD)		15.0 (1.7)	15.1 (1.5)	14.9 (1.8)
Full-time student, n (%)		43 (48)	27 (45)	16 (53)

**Table 2:** Ratings of therapist and brief motivational interviewing traits by intervention.

Traits		Computer-delivered intervention, mean (SD)		*t*_76_	*P*	α
		Voice	Text			
**Therapist traits^a^**				0.882	.46	.89
	How engaging was the system?	4.6 (1.4)	4.3 (1.7)			
	How empathetic was the system?	3.7 (1.5)	4.3 (1.6)			
	How warm was the system?	3.8 (1.5)	4.0 (1.7)			
	How well did the system understand you?	4.4 (1.6)	4.5 (1.9)			
	How satisfied did you feel with the system?	4.3 (1.5)	4.7 (1.6)			
	Total	4.2 (1.2)	4.4 (1.5)			
**Brief motivational interviewing traits^b^**				–0.555	.48	.83
	Was easy to interact with	3.0 (0.6)	3.2 (0.6)			
	Understood me	2.8 (0.7)	2.8 (0.7)			
	Asked about my ideas before presenting its own	3.2 (0.5)	2.9 (0.6)			
	Helped me talk about my own reasons for change	3.1 (0.6)	2.8 (0.7)			
	Respected my ideas about how I might make changes	3.1 (0.5)	2.9 (0.6)			
	Did not push me into something I wasn’t ready for	3.1 (0.6)	3.0 (0.5)			
	Accepted that I might not want to change	3.0 (0.7)	2.9 (0.7)			
	I felt engaged in the session (willing to discuss drinking)	3.1 (0.7)	3.0 (0.6)			
	Total	3.0 (0.4)	2.9 (0.5)			

^a^ Five items rated on a 7-point Likert scale (1=“not at all” to 7=“very”).

^b^ Eight items rated on a 4-point Likert scale (1=“strongly disagree” to 4=“strongly agree”).

### Drinking-Related Outcomes

Attrition analyses were conducted to assess if there were any significant differences between participants who completed the follow-up assessment and those who did not. Noncompleters were not significantly different from completers in terms of demographics, number of drinks consumed per week, or number of heavy drinking episodes in past month. However, a significant difference was observed between completers and noncompleters in number of alcohol-related problems (BYAACQ), with noncompleters (n=12; voice: n=8, 13%; text: n=4, 13%) endorsing significantly more alcohol-related problems at baseline (mean difference 2.94; *t*_87_=–2.195, *P*=.03). The baseline BYAACQ score for noncompleters was not significantly different between conditions (*t*_10_=0.69, *P*=.51).

Paired *t* tests showed significant main effects of time indicating reductions in drinks consumed per week (*t*_51_=–3.56, *P*=.001), number of heavy drinking days (*t*_51_=–4.53, *P*<.001), and reported problems with alcohol use (*t*_51_=–3.60, *P*=.001) from baseline to the 1-month follow-up assessment in the voice-based computer-delivered intervention condition. Participants in the voice-based computer-delivered intervention condition also reported a significant increase in importance of changing drinking (*t*_49_=2.60, *P*=.01) from baseline to 1-month follow-up; however, no significant change was observed in reported confidence to change drinking behaviors (*t*_51_=1.47, *P*=.15; see Table 3 for means and SDs).

**Table 3:** Baseline and follow-up alcohol-related measures for the full sample and by condition.

Variable	Full sample, mean (SD) (N=90)		Voice-based computer-delivered intervention, mean (SD) (n=60)		Text-based computer-delivered intervention, mean (SD) (n=30)	
	Baseline	Follow-up	Baseline	Follow-up	Baseline	Follow-up
Number of drinks per week^a^	10.1 (8.2)	7.5 (6.0)	9.6 (7.2)	7.0 (5.4)	11.2 (9.9)	8.6 (7.0)
Number of heavy drinking days^a^	4.4 (3.5)	2.8 (2.7)	4.3 (3.3)	2.7 (2.8)	4.6 (3.9)	2.9 (2.5)
Alcohol-related problems^a^	6.1 (4.3)	4.9 (4.2)	5.9 (4.3)	4.0 (3.3)	6.5 (4.1)	6.8 (5.2)
Importance of changing drinking	2.7 (2.2)	3.5 (3.0)	2.5 (2.0)	3.6 (3.1)	3.0 (2.5)	3.3 (2.9)
Confidence to change drinking	7.7 (2.1)	8.3 (1.9)	7.8 (2.1)	8.4 (1.8)	7.7 (2.2)	8.2 (2.3)

^a^ Number of drinks per week and number of heavy drinking days in the past month were collected via Alcohol Timeline Follow-back. Alcohol-related problems experienced in the past month were assessed via BYAACQ.

Covarying baseline alcohol-related problems, participants randomized to the voice-based computer-delivered intervention reported 40% fewer alcohol-related problems at follow-up compared to participants in the text-based condition (incident rate ratio [IRR]=0.60, 95% CI 0.44-0.83, *P*=.002). Experimental condition did not significantly predict number of drinks consumed per week (B=–0.12, 95% CI –0.41 to 0.17, *P*=.41), number of heavy drinking days (IRR 1.07, 95% CI 0.75-1.53, *P*=.72), or rated importance of changing drinking (B=0.76, 95% CI –0.51 to 2.03, *P*=.24) at the 1-month follow-up, covarying for the respective dependent variable at baseline.

## Discussion

This study represents a promising initial step toward developing a computer-delivered intervention for heavy drinking that relies on an interactive voice-based system rather than a traditional keyboard-and-mouse text-based system. Results showed that it was feasible to create a set of predetermined questions and responses that were sufficient to direct a user through the typical components of a brief MI, while demonstrating to users that their responses were heard and understood. Participants receiving the voice-based computer-delivered intervention agreed that the system demonstrated MI-consistent behavior (eg, helped me talk about reasons for change, asked me about my ideas before presenting its own), and displayed at least moderate levels of general therapist traits (eg, was understanding, was engaging). When compared to a text-based computer-delivered intervention, the voice-based computer-delivered intervention appeared to perform equally well in terms of these system ratings. Although no significant differences on the total score for either scale were observed between conditions, several ratings on the individual-item level that might have been expected to be greater for the voice-based computer-delivered intervention were observed to be numerically lower than the text-based computer-delivered intervention; for example, empathy and warmth were rated lower on average for the voice-based computer-delivered intervention. The observation that the point-and-click interface (text-based computer-delivered intervention) may be rated at least as, if not more, empathetic/warm than the voice-based computer-delivered intervention highlights potential areas for improvement. We speculate that the voice we used for the voice-based computer-delivered intervention system, which had a distinctly robotic tone, may have contributed to these relatively low user ratings. Furthermore, we did not have an onscreen avatar or other visual presence during the session, and some participants expressed, while interacting with the voice-based computer-delivered intervention, that they were unsure whether they should be speaking to the static image on the computer screen or looking elsewhere.

Participants in the voice-based computer-delivered intervention condition reported significant decreases in number of drinks consumed and number of heavy drinking days, and significant increases in perceived importance of changing drinking, but confidence in their ability to change drinking, which was high at baseline, did not increase significantly. The voice-based computer-delivered intervention, compared to the text-based computer-delivered intervention, did not result in significantly greater change on any of these variables, and the differences between the conditions on these variables were small. However, we did observe a significant difference between conditions in alcohol-related problems reported at 1-month follow-up. Specifically, those randomized to the voice-based computer-delivered intervention, compared to those in the text-based computer-delivered intervention, reported about a 40% lower number of alcohol problems in the month after intervention. Although drinking was reduced following both computer-delivered interventions, only the voice-based computer-delivered intervention appeared to lead to a reduction in alcohol problems.

The fact that the voice-based computer-delivered intervention, compared to the text-based computer-delivered intervention, resulted in significantly lower alcohol-related problems but did not appear to have a greater effect on reducing alcohol consumption was unexpected. However, previous studies have demonstrated that alcohol consumption and problems have distinct etiological pathways [35,36], and may not respond to intervention in parallel. Moreover, two previous studies examining face-to-face MIs have demonstrated intervention effects for reducing alcohol problems in the absence of reducing alcohol consumption [37,38]. It may be that verbalizing the problems experienced due to drinking may help individuals think forward to potential problems and be more aware of the need to protect against these. However, future studies are required to identify specific factors that account for the differences in alcohol-related problems observed between the voice-based and text-based computer-delivered interventions.

Several important limitations should be taken into consideration when evaluating results of this study. First, the sample consisted of college-aged participants who met criteria for heavy drinking, but whose overall levels of drinking were relatively low compared to other intervention studies with college students (eg, [39]); thus, these results may not generalize to non-college-aged populations or heavier drinking college populations. Second, participants were aware that their responses to the voice-based computer-delivered intervention were being audio-recorded and were audible to the research assistant, which may have made them feel less comfortable in the interaction. Third, this study compared voice-based computer-delivered intervention to a text-based computer-delivered intervention that followed the same intervention content and outline rather than to an existing empirically supported text-based computer-delivered intervention. This comparison was to allow us to determine experimentally whether the difference in delivery format was acceptable to participants. Results of this study are not intended to support the efficacy of the voice-based computer-delivered intervention relative to an established intervention. The human-controlled voice-based computer-delivered intervention was developed as a proof of concept and should not be considered an ecologically valid or practical health intervention in its present form. The voice-based computer-delivered intervention was only compared to the text-based computer-delivered intervention rather than a human interventionist. Therefore, the fact that acceptability ratings were equal between the voice-based computer-delivered intervention and the text-based computer-delivered intervention may overestimate the system’s performance on these ratings relative to comparing them to a human interventionist. Fourth, in regard to the changes reported in alcohol-related behaviors, we are unable to evaluate the effect of assessment reactivity on outcomes; it is possible that completing the laboratory-based assessment and Web-based follow-up assessment may have influenced participants’ alcohol-related behaviors and accounts for the reductions in drinking we observed [40]. Finally, the study was powered to detect medium-sized reductions in alcohol use and problems within the voice-based computer-delivered intervention condition rather than to test differences relative to the text-based computer-delivered intervention. Therefore, analyses comparing the voice-based computer-delivered intervention to the text-based computer-delivered intervention should be considered preliminary.

The task of constructing a voice-based computer-delivered intervention that can ask questions about alcohol use and respond in a manner consistent with MI practice is a challenging one. First, the voice-based computer-delivered intervention used in this study relied on a human controller. We have recorded participant responses and therefore can analyze the participant verbal behavior that led to specific choices by the human controller about which response button to push. Machine-learning algorithms may be able to detect the key verbal content and configurations that suggest the appropriate response, which can then be used to develop a prototype of an automated system.

Prior research has shown that people respond more strongly to automated systems that are more emotive in speech and animation. For example, users tasked with training a robot how to dance trained with the robot longer and with more accurate examples when the robot’s reactions to its progress were more emotive [41]. For similar reasons, it will be important to examine modifications to the voice-based computer-delivered intervention system that may help to increase therapist and MI ratings. For example, the current voice-based computer-delivered intervention system used a standard computerized voice (macOS VoiceOver); voices that better approximate natural human speech may increase user acceptability ratings, particularly those that reflect human traits (warmth, empathy).

The use of a voice-based system that can allow for greater personalization of the computerized interventionist (eg, allowing the system to introduce itself and address the participant directly) may help to increase general therapist ratings. The system could also be made more sophisticated by creating ways in which information obtained earlier in the interaction are reintroduced later in the interaction, such as when the user is making a change plan. This would be particularly important in regards to change talk, which could be reiterated in later portions of the session to make it more salient to the user. Identifying mediating variables that account for the differences observed between the interventions will help inform future directions for improving the voice-based computer-delivered intervention. In particular, it would be useful to know what strategies participants used to avoid alcohol-related problems. That information could be used, in turn, to improve the voice-based computer-delivered intervention by highlighting those potential strategies when completing a change plan. Finally, an emerging line of experimental research has shown that compared to screen avatars, embodied robots (ie, robots that have a physical form and are in the room with participants) elicit greater engagement and compliance from people who are following directions from the automated system [42,43]. Embodying the computer-delivered intervention in a robot may be a powerful means of increasing participants’ perceptions of the computer-delivered intervention’s empathy and warmth and may increase overall engagement with the system.

## Abbreviations

BYAACQ: Brief Young Adult Alcohol Consequences Questionnaire
IRR: incident rate ratio
MI: motivational interviewing
PC: personal computer
